# SEH1L siliencing induces ferroptosis and suppresses hepatocellular carcinoma progression via ATF3/HMOX1/GPX4 axis

**DOI:** 10.1007/s10495-024-02009-5

**Published:** 2024-08-02

**Authors:** Ziyang Feng, Ke Cao, Haojia Sun, Xuewen Liu

**Affiliations:** 1 Postdoctoral Station of Medical Aspects of Specific Environments, The Third Xiangya Hospital, Central South University, Changsha, 410013 Hunan P.R. China; 2 Department of Oncology, The Third Xiangya Hospital, Central South University, Changsha, 410013 Hunan P.R. China

**Keywords:** SEH1L, HCC, Ferroptosis, ATF3, HMOX1

## Abstract

SEH1 like nucleoporin (SEH1L) is an important component of nuclear pore complex (NPC), which is crucial in the regulation of cell division. However, the interrelation between SEH1L expression and tumor progression is less studied. In this research, we performed a systematic bioinformatic analysis about SEH1L using TCGA, Timer 2.0, Cbioportal, UCLAN and CellMiner™ databases in pan-cancer. Besides, we further validated the bioinformatic results through in vitro and in vivo experiments in HCC, including transcriptome sequencing, real-time quantitative PCR (RT-qPCR), western blotting (WB), immunohistochemistry (IHC), cell proliferation assays, clone formation, EdU, transwell, flow cytometry and subcutaneous tumor model. Our results suggested that SEH1L was significantly up-regulated and related to poor prognosis in most cancers, and may serve as a potential biomarker. SEH1L could promote HCC progression in vitro and in vivo. Besides, the next generation sequencing suggested that 684 genes was significantly up-regulated and 678 genes was down-regulated after the knock down of SEH1L. SEH1L siliencing could activate ATF3/HMOX1/GPX4 axis, decrease mitochondrial membrane potential and GSH, but increase ROS and MDA, and these effects could be reversed by the knock down of ATF3. This study indicated that SEH1L siliencing could induce ferroptosis and suppresses hepatocellular carcinoma (HCC) progression via ATF3/HMOX1/GPX4 axis.

**Supplementary Information:**

The online version contains supplementary material available at 10.1007/s10495-024-02009-5.

## Introduction

Currently, cancer has become a major reason of disease-related death in humans [1, 2]. Among them, the mortality of liver hepatocellular carcinom (LIHC) ranks second in the world [3–5]. The etiology of LIHC includes infection, alcohol, aflatoxin, cirrhosis, and heredity [6, 7]. Currently, LIHC can be divided into hepatocellular carcinoma (HCC), hepatobiliary epithelial carcinoma and mixed hepatocellular carcinoma [8, 9]. In LIHC patients, HCC accounting for over 90% and is the most common type [10]. Recently, the treatment of HCC has achieved great progress, including surgery, chemotherapy, radiotherapy, target therapy, interventional therapy and immunotherapy [11–13]. However, many HCC patients were diagnosed at the advanced stages, the five-year overall survival rate (OS) of HCC is just 18% [14, 15]. Therefore, it is of great importance to find new diagnostic and therapeutic targets for HCC.

Ferroptosis is a new type of iron dependent cell death, which is different from apoptosis and autophagy [16, 17]. Ferroptosis is present in inflammation, ischemia-reperfusion injury, acute kidney injury and tumors, and plays important roles in disease process [18]. The occurrence of ferroptosis is closely related to lipid peroxidation, abnormal iron metabolism, glutathione (GSH) depletion and glutathione peroxidase 4 (GPX4) inactivation [19]. GPX4 is an important antioxidant enzyme, and can directly reduce lipid peroxides and play an important role in the regulation of reactive oxygen species (ROS) [20]. In recent years, many studies have suggested that ferroptosis participates in the regulation of HCC progression. For instance, Yimin Zheng et al. found that inhibition of PGAM1 could promote HCC ferroptosis and synergize with anti-PD-1 immunotherapy [21]. Feng He et al. found that ATF4 suppresses hepatocarcinogenesis by inducing SLC7A11 to block stress-related ferroptosis [22]. However, the role of ferroptosis in HCC is quite complex and need to be further studied.

SEH1 like nucleoporin (SEH1L), part of a nuclear pore complex (NPC), plays important roles in mTORC1 regulation as an inhibitor of the Rag GTPases [23]. Besides, studies have shown that SEH1L participated in the regulation of cell division by regulating chromosome alignment and segregation [24]. Recently, several studies reported that SEH1L may function as a biomarker in cancer. Jing Yu et al. found that SEH1L was down-regulated in ovarian cancer and may function as a prognostic biomarker [25]. Nevertheless, the function of SEH1L in cancer is still in need of thorough research.

In our study, we made a comprehensive analysis about SEH1L, and found that it was significantly up-regulated in pan-cancer and may function as a biomarker. Knock down of SEH1L could suppress HCC progression in vitro and in vivo. Furthermore, the next generation sequencing was performed to explore the down-stream targets. SEH1L siliencing could activate ATF3/HMOX1/GPX4 pathway, decrease mitochondrial membrane potential and GSH, but increase ROS and MDA, and these effects could be reversed by the knock down of ATF3. This study indicated that SEH1L siliencing could induce ferroptosis and suppresses HCC progression via ATF3/HMOX1/GPX4 axis.

## Materials and methods

### SEH1L expression, diagnostic and prognostic value analysis

The expression of SEH1L was analyzed using Timer (timer.cistrome.org) database, the “P values” was set as “0.05”, the “folding change” was set as “1.5”. The diagnostic value of SEH1L was detected using ROC curves with TCGA data (https://www.cancer.gov/ccg/research/genome-sequencing/tcga). The prognostic value of SEH1L was detected using overall survival (OS), disease specific survival (DSS), disease-free interval (DFI), and progression-free interval (PFI) with TCGA data. The correlation was analyzed using the “pROC” and “ggplot2” R packages.

### SEH1L mutation, methylation and drug sensitivity analysis

The mutation characteristic of SEH1L was analyzed using cBioPortal tool (https://www.cbioportal.org/). The mutation sites and types of SEH1L was detected in the “cancer type summary” module. The “comparison/survival” module was used to reveal the correlation between SEH1L mutation and prognosis.

The methylation level of SEH1L was analyzed using UCLAN database (https://ualcan.path.uab.edu/) with TCGA data.

The mRNA expression of SEH1L and IC50 values of different drugs were obtained from the CellMiner™ database (https://discover.nci.nih.gov/cellminer/home.do). Simultaneously, the correlation scatterplots were drew using the “impute”, “ggplot2” and “limma” R packages.

### Next generation sequencing

MHCC97H cells were transfected with SEH1L-si RNA, the total RNA of control group and knock down group were extracted using Trizol (Accurate Biology, China). The sequencing were performed by Allwegene Technologies (Beijing, China). The differential expressed genes was analyzed with EdgeR version 3.08. Benjamini- Hochburg method was used to calculate the adjusted P values. The threshold was adjusted *P* < 0.05.

### HCC tissues collection

All the HCC tumors and paired adjacent noncancerous tissues were collected from 16 patients who were diagnosed with HCC and had undergone surgery at the Third Xinagya Hospital, Central South University, Changsha, Hunan, P.R. China. Histologic and pathologic diagnoses of tissue samples were independently confirmed by 2 experienced histopathologists. The HCC tissues were collected after the surgery and stored at -80 °C. All the patients had signed the informed consent. The study was approved by the Third Xiangya Hospital Ethics Committee, and the procedures were conducted according to the Declaration of Helsinki.

### Cell lines and cell culture

All the cell lines, including HCCLM3, MHCC97H, PLC/PRF/5, Huh7 and LO2 cells were purchased from the Institutes of Biomedical Sciences. HCCLM3, MHCC97H and Huh7 were cultured with DMEM medium, PLC/PRF/5 was cultured with MEM medium, and LO2 was cultured with 1640 medium. The medium was added with 10% fetal bovine serum (BS-1105, Inner Mongolia Opcel Biotechnology Co.,Ltd., China) and 1% penicillin/streptomycin. The cells were cultured in a humidified incubator at 37 ℃, in an atmosphere of 5% CO2.

### Real-time quantitative PCR (RT-qPCR)

The total RNA was extracted using TRIZOL (Accurate Biology, China). After measuring the concentration of RNA, 1 µg total RNA was used to perform the reverse transcription using the RT Kit with gDNA Clean for qPCR (Accurate Biology, China). Then the product of reverse transcription was diluted 10 times with deionized water. Finally, RT-qPCR was performed using the Premix Pro Taq HS qPCR Kit (Accurate Biology, China) on ViiA™ 7 RT-PCR system. All the primers are summarized as follows: β-Actin-F: CTCCATCCTGGCCTCGCTGT, β-Actin-R: GCTGTCACCTTCACCGTTCC; SEH1L-F: TCTTCTGCTGGCAGGTATTTCT, SEH1L-R: AGTGTCAGCATCGCAAGAGT. ATF3-F: CTCGGGGTGTCCATCACAAA, ATF3-R: GGCACTCCGTCTTCTCCTTC; CA9-F: GGCTACAGCTGAACTTCCGA, CA9-R: TGACAGCAAAAAGGAGGCCA; CP-F: CTCACAATGCACGTGGGAGA, CP-R: CAGCCAGATTTGGTGTCTTCATTT; HMOX1-F: ACTCCCTGGAGATGACTCCC, HMOX1-R: TCTTGCACTTTGTTGCTGGC, NR1D-F: CAACACAGGTGGCGTCATCA, NR1D-R: CTGGAAGCTGCCATTGGAGT; SCD-F: CTTGCGATATGCTGTGGTGC; SCD-R: CCGGGGGCTAATGTTCTTGT; TNFAIP3-F: TCCACAAAGCCCTCATCGAC, TNFAIP3-R: TTCGTTTTCAGCGCCACAAG.

### Western blotting (WB)

The proteins in tissues and cells were extracted using RIPA (Beyotime, China) with 10% Protease and Phosphatase Inhibitor (NCM biotech, China). After measuring the concentration of protein, 30 µg proteins were electrophoresed under 80 V for 30 min and then 120 V for 60 min. Then, the gel was transferred to the PVDF membranes under 250 mA for 100 min. Subsequently, the membranes were blocked with 5% non-fat milk at room temperature for 60 min. Then the membranes were incubated with a primary antibody at 4 ℃ overnight. The next day, the membranes were washed three times with TBST and incubated with a secondary antibody at room temperature for 60 min. Finally, the blots were visualized using the ChemiDocXRS + System (Bio-Rad, America). The antibodies used are β-Actin (Proteintech, China), SEH1L (Proteintech, China), ATF3 (Abcam, America), HMOX1 (AiFang Biological, China), GPX4 (AiFang Biological, China).

### Immunohistochemistry

At first, the sections were dewaxed and dehydrated using xylene and ethanol. Then the sections were boiled with citrate for 10 min. Next, the sections were incubated with 3% H2O2 at room temperature for 10 min. After blocking with goat serum, the sections were incubated with primary antibodies overnight at 4 ℃. The next day, the sections were washed three times with PBST at room temperature and incubated with a secondary antibody at room temperature for 30 min. Finally, the sections were stained with DAB and hematoxylin, and an N2-Mi8 microscope was used to capture the images.

### Cell proliferation assays

At first, 10% CCK-8 (Biosharp, China) were added to the 96-well cells. Then, the cells were cultured in 37 °C for 180 min. Finally, the Microplate Reader was used to detect the absorbance at 450 nm.

### Clone formation

The cells were seeded into a 6-well plate at a concentration of 500 cells/well. The medium was replaced every three days. At about two weeks later, the cells were fixed using 4% paraformaldehyde at room temperature for 30 min. Then, the cells were stained with 0.1% crystal violet for 30 min.

### Transwell

At first, 50,000 cells were seeded into the upper chamber with 200 µl medium (1% serum). Then, 600 µl medium (20％ serum) were added into the lower chamber. About 24 h later, the cells of the upper chamber were removed, and the cells of the lower chamber were fixed with 4% paraformaldehyde for 20 min. Then, the cells were stained with 0.1% crystal violet for 30 min. The N2-Mi8 microscope was used to capture the images.

### EdU

The cells were seeded into a 96-well plate at a concentration of 10,000 cells/well. The next day, the cells were incubated with the EdU solution (1:1000) for 2 h. Then, the cells were fixed with 4% paraformaldehyde for 20 min. Next, the cells were incubated with the Apollo dye solution (RiboBIO, China) for 30 min. Then, the cells were washed with 0.5% TritonX-100 for 10 min. Finally, the cells were incubated with the DAPI solution at room temperature for 10 min. The N2-Mi8 microscope was used to capture the images.

### ROS detection

ROS was detected using ROS Assay Kit (Beyotime, China). The cells were seeded in the 6-well plates and cultured overnight. The next day, DCFH-DA was diluted 1000 times using DMEM. Then the cells were incubated for 60 min. Subsequently, the cells were washed with DMEM for 3 times, and then collecting the cells. Flow cytometry was used to detect the ROS level.

### Mitochondrial membrane potential detection

Mitochondrial membrane potential was detected using Rhodamine 123 (Beyotime, China). The cells were seeded in the 24-well plates and cultured overnight. The next day, Rhodamine 123 was diluted 1000 times using the buffer. Then the cells were incubated for 60 min. Subsequently, the cells were washed with PBS for 3 times, and then collecting the cells. The N2-Mi8 microscope was used to capture the images.

### GSH detection

GSH was detected using GSH and GSSG Assay Kit (Beyotime, China). The cells were seeded in the 6-well plates and cultured overnight. The next day, collect the cells and re-suspend the cells in 30 µL of solution M. Then the cells were lysed using liquid nitrogen and 37 °C water. Collect the supernatant after centrifugal at 10, 000 rpm, 4 °C. Then prepare the detection working solution according to the instructions and incubate at 25 °C for 5 min. Subsequently, add 50 µL NADPH and incubate for 25 min, measure the absorbance at 412 nm.

### MDA detection

MDA was detected using Lipid Peroxidation MDA Assay Kit (Beyotime, China). The cells were seeded in the 6-well plates and cultured overnight. The next day, the cells were lysed and collect the supernatant. The reaction system was mixed according to instructions. Then, the mixture was boiled at 100 °C for 15 min. Then collect the supernatant after centrifugal at 1, 000 g, room temperature. Measure the absorbance at 532 nm.

### In vivo xenograft studies

For the subcutaneous tumor model, the male BALB/C nude mice (4 weeks old, weight 16 g) were divided into 2 groups. 5 × 10^6^ cells were injected subcutaneously into the armpit. The tumor size was measured every three days. Three weeks later, the mice were sacrificed. The expression of genes was detected using IHC.

The animal experiments were approved by the Ethics Committee for laboratory animal of Central South University.

### Statistical analysis

The statistical analysis was performed using GraphPad Prism. Student’s t-test was used to compare the difference between two groups, and a one-way analysis of variance was used to compare the difference between multiple groups. The data obtained are shown as average values ± SD. *P* < 0.05 was considered statistically significant.

## Results

### The expression of SEH1L in human tissues and cancers

**Fig. 1:**
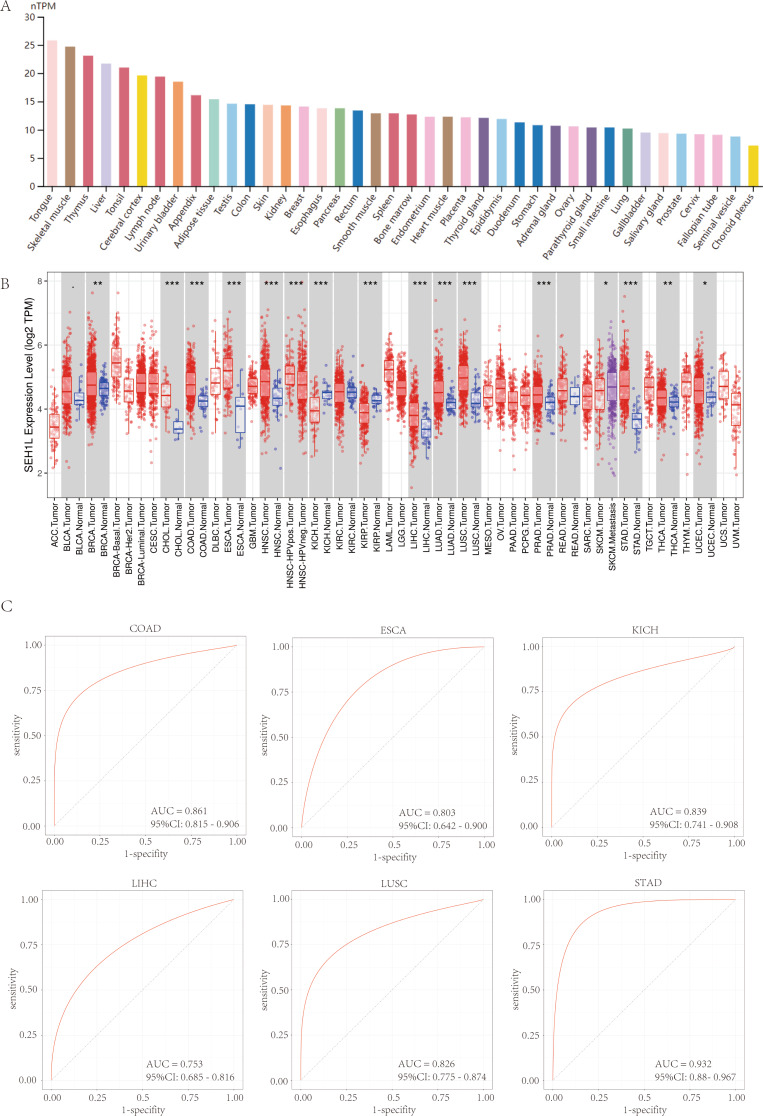
The mRNA expression level of SEH1L in normal tissue and pan-cancer. (**A**) The expression of SEH1L in normal tissue. (**B**) SEH1L was up-regulated in most cancer (**C**) The ROC curves of SEH1L in COAD, ESCA, KICH, LIHC, LUSC and STAD

At first, we detected the expression level of SEH1L in HPA database. In normal human tissues, SEH1L was highly expressed in tongue, skeletal muscle, thymus, liver and tonsil, but was lowly expressed in choroid plexus, seminal vesicle, fallopian tube, cervix and prostate (Fig. 1A). Then, the expression of SEH1L in pan-cancer was analyzed using Timer 2.0 database. The results suggested that SEH1L was significantly up-regulated in most human cancers, including breast invasive cancer (BRCA), cholangiocarcinoma (CHOL), colon adenocarcinoma (COAD) and so on. By contrast, SEH1L was down-regulated in kidney chromophobe (KICH) and kidney papillary cell carcinoma (KIRP) (Fig. 1B).

Next, we assessed the diagnostic value of SEH1L in pan-cancer using ROC curve. As illustrated in Fig. 1C, SEH1L can serve as a potential diagnostic biomarker in COAD, esophageal carcinoma (ESCA), KICH, LIHC, lung squamous cell carcinoma (LUSC) and stomach adenocarcinoma (STAD) (AUC: 0.861, 0.803, 0.839, 0.753, 0.826 and 0.932 respectively).

### The prognostic value of SEH1L in pan-cancer

**Fig. 2:**
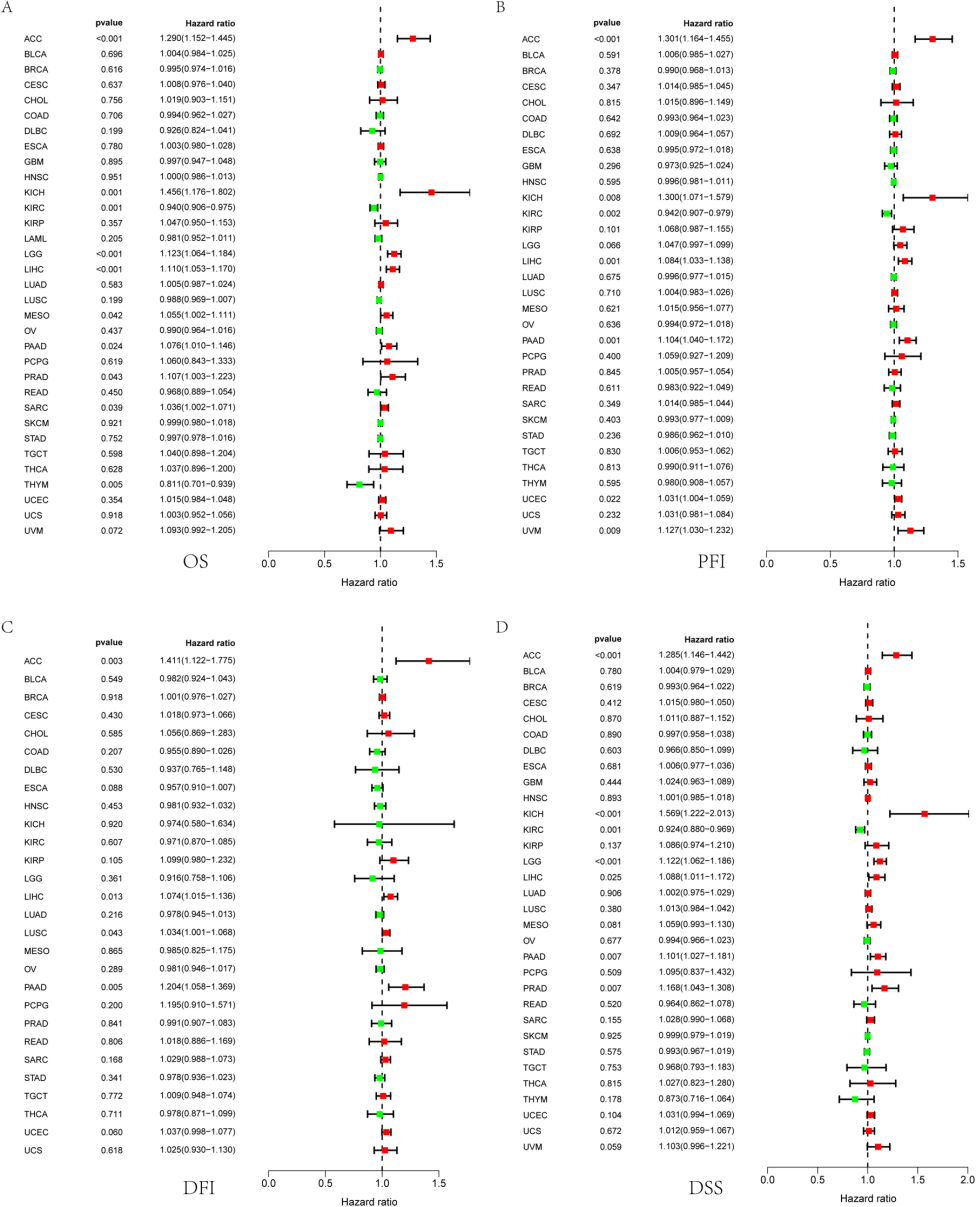
SEH1L may function as a prognostic biomarker in pan-cancer. (**A**) Overall survival. (**B**) Progression free interval. (**C**) Disease free interval. (**D**D) Disease specific survival

Based on the TCGA database, we further analyzed the prognostic value of SEH1L in pan-cancer. High expression of SEH1L was associated with poor OS in adrenocortical carcinoma (ACC), KICH, brain lower grade glioma (LGG), LIHC, mesothelioma (MESO), pancreatic adenocarcinoma (PAAD), prostate adenocarcinoma (PRAD) and sarcomav (SARC) (Fig. 2A). High SEH1L expression was a risk factor for shorter progression free interval (PFI) in ACC, KICH, LIHC, PAAD, uterine corpus endometrial carcinoma (UCEC) and uveal melanoma (UVM) (Fig. 2B). Up-regulation of SEH1L may shorten the disease free interval (DFI) of ACC, LIHC and PAAD (Fig. 2C). Besides, high SEH1L expression was correlated with shorter disease specific survival (DSS) in ACC, KICH, LGG, LIHC, PAAD and PRAD (Fig. 2D).

### The mutation and methylation characteristic of SEH1L in pan-cancer

The mutation characteristics of SEH1L in pan-cancer was analyzed using cBioPortal database. Highest frequency of SEH1L alteration (9.52%) occurred in uterine mixed endometrial carcinoma patients (Figure S1). Figure S2A showed the alteration sites, types and numbers of SEH1L in pan-cancer. Next, we analyzed the correlation between SEH1L mutation and cancer prognosis. We found that UCEC patients with SEH1L mutation had better prognosis in OS (*P* = 0.0324), DSS (*P* = 0.0311) and PFS (*P* = 0.0431) (Figure S2B-E).

**Fig. 3:**
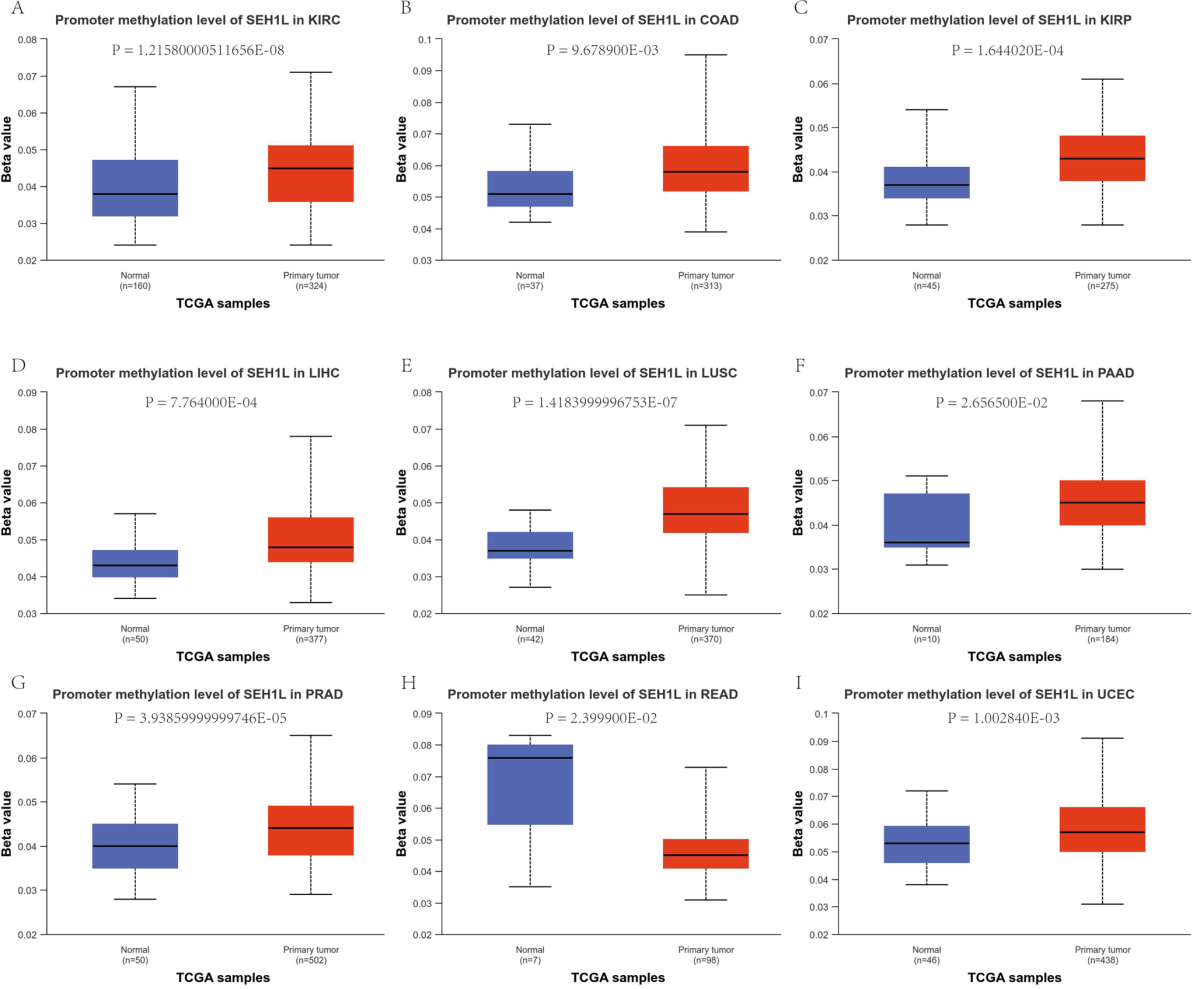
The promoter methylation characteristic of SEH1L in pan-cancer. (**A**-**I**) The promoter methylation level of SEH1L in KIRC, COAD, KIRP, LIHC, LUSC, PAAD, PRAD, READ and UCEC

Then, we evaluated the methylation level of SEH1L promoter between cancer and normal tissue. The results suggested that the methylation level was up-regulated in KIRC, COAD, KIRP, LIHC, LUSC, PAAD, PRAD and UCEC, but was down- regulated in READ (Fig. 3A-I).

### The drug sensitivity analysis of SEH1L

**Fig. 4:**
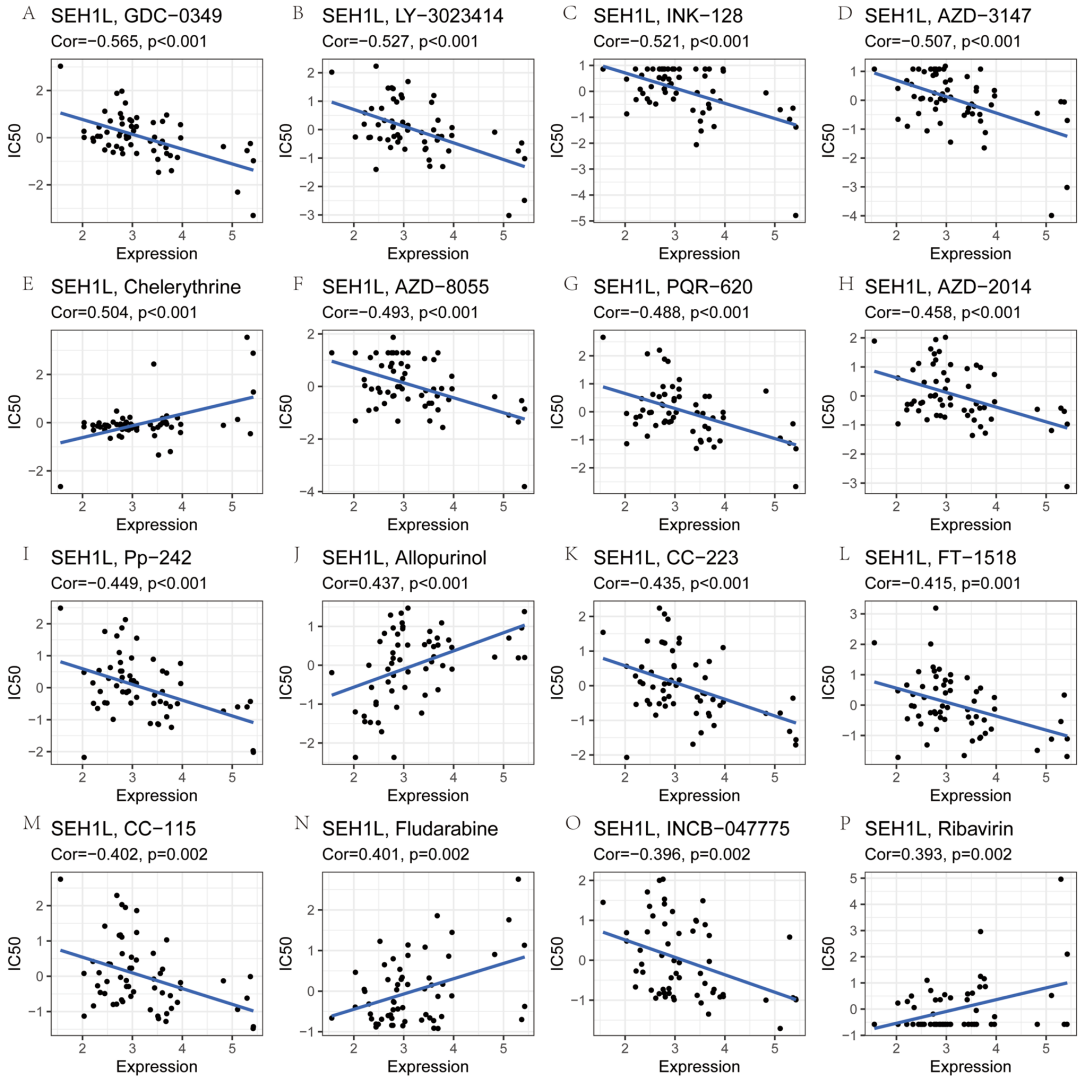
The drug sensitivity analysis of SEH1L. (**A**-**D**, **F**-**I**, **K**-**M**, **O**) A negative correlation was shown between SEH1L expression and drug IC50. (**E**, **G, N, P**) A positive correlation was shown between SEH1L expression and drug IC50

Next, the correlation between SEH1L expression and drug sensitivity was analyzed using CellMiner database. The expression of SEH1L was positively related to the drug sensitivity of chelerythrine, allopurinol, fludarabin and ribavirin. However, SEH1L was negatively associated with the sensitivity of GDC-0349, LY-3,023,414, INK-128, AZD-3147, AZD-8055, PQR-620, AZD-2014, Pp-242, CC-223, FT-1518, CC-115 and INCB-047775 (Fig. 4A-P).

### Knock down of SEH1L induced ferroptosis and suppressed HCC progression

**Fig. 5:**
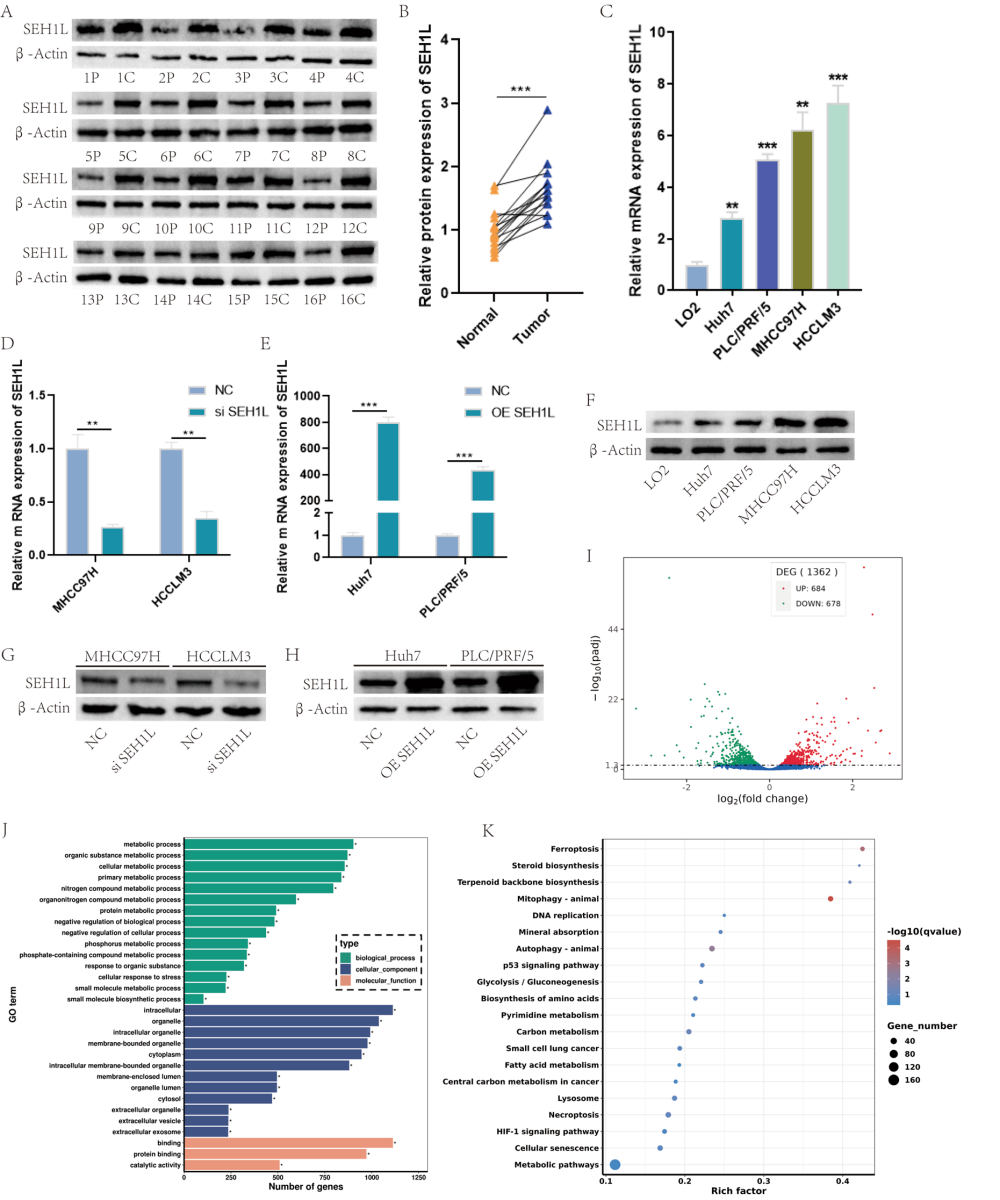
SEH1L was up-regulated in HCC. (**A**-**B**) The protein expression of SEH1L in HCC (C: cancer P: paracancerous non-cancer). (**C**) The mRNA expression of SEH1L in HCC cells. (**D**-**E**) The SEH1L mRNA knock down and over-expression efficiency. (**F**) The protein expression of SEH1L in HCC cells. (**G**-**H**) The SEH1L protein knock down and over-expression efficiency. (**I**) Next generation sequencing showed 684 up-regulated genes and 678 down-regulated genes after SEH1L knock down. (**J**-**K**) GO and KEGG analysis of the different expressed genes

In above section, we made a comprehensive analysis about the expression level, diagnostic value, prognostic value, mutation and methylation characteristic of SEH1L in pan-cancer. The results suggested that SEH1L was significantly up-regulated in HCC, and may function as a potential diagnostic and prognostic biomarker in HCC. Thus, we further validated the bioinformatic results through in vitro and in vivo experiments in HCC. At first, we detected the expression of SEH1L in HCC cell lines and cancer tissues. Figure 5A-B suggested that SEH1L was up-regulated in HCC cancer tissues. Besides, the RNA and protein expression level was also elevated in HCCLM3, MHCC97H, PLC/PRF/5 and Huh7 cell lines (Fig. 5C, F).

Next, to further analyze the function of SEH1L, SEH1L was knocked down and over-expressed in HCC cells lines (Fig. 5D-E, G-H). The next generation sequencing of MHCC97H cells suggested that after the knock down of SEH1L, 684 genes was significantly up-regulated and 678 genes was down-regulated (Fig. 5I). The GO analysis suggested that SEH1L joined in the regulation of metabolic process, organic substance metabolic process (biological_process), intracellualr, organelle (cellular_component), binding and protein binding (molecular_function) (Fig. 5J). KEGG analysis illustrated the enrichment of ferroptosis, steroid biosynthesis and terpenoid backbone biosynthesis (Fig. 5K).

**Fig. 6:**
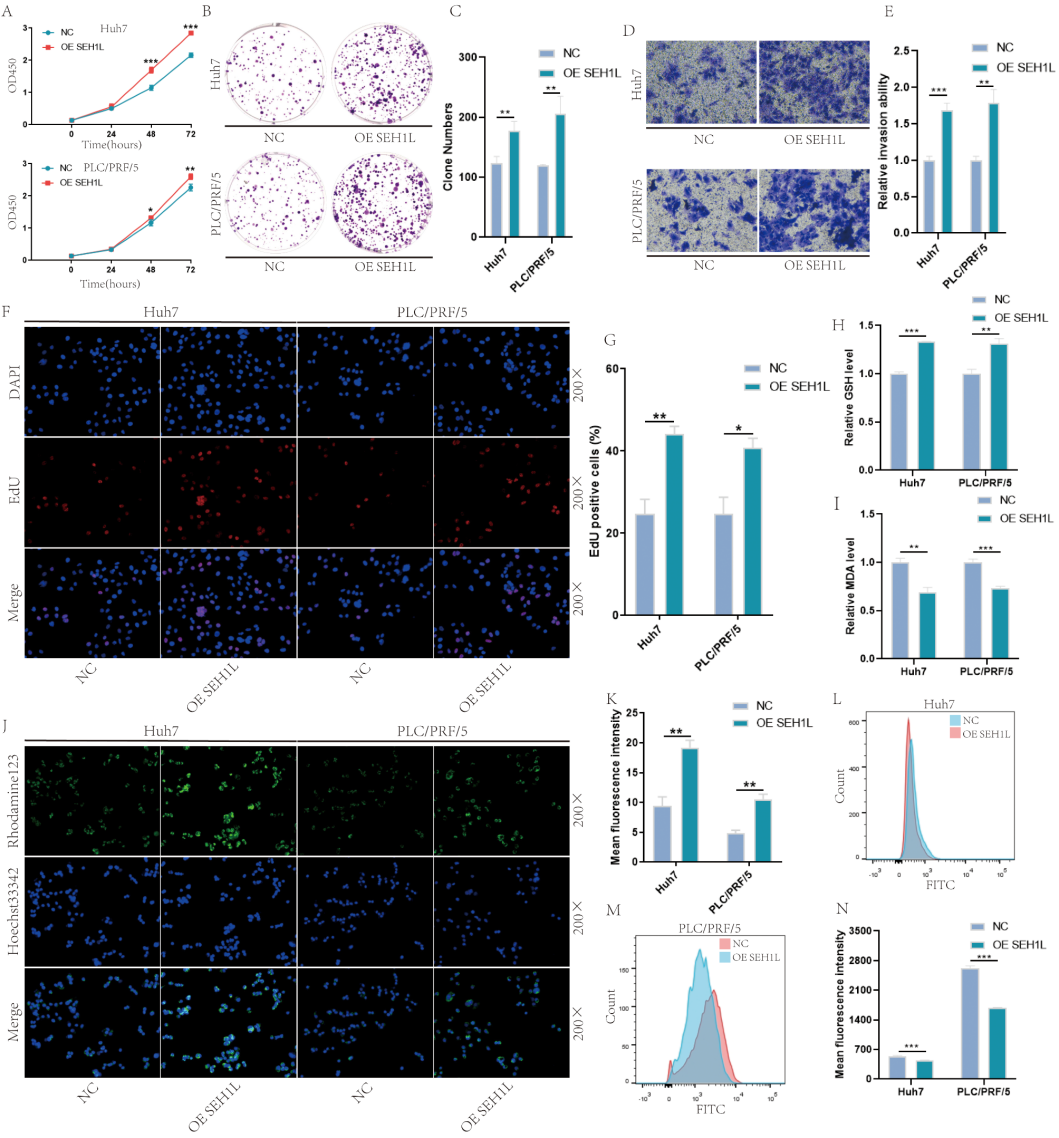
Over-expression of SEH1L promoted HCC progression. (**A**-**C**, **F**-**G**) SEH1L promoted the proliferation of HCC. (D-E) SEH1L promoted HCC migration. (**H**-**I**) Over-expression of SEH1L increased HCC GSH, but decreased MDA. (**J**-**K**) Over-expression of SEH1L increased HCC mitochondrial membrane potential. (**L**-**N**) Over-expression of SEH1L decreased HCC ROS. (* represents *P* < 0.05, ** represents *P* < 0.01, *** represents *P* < 0.001)

Then, we detected the influence of SEH1L in HCC progress. The results of CCK8, clone formation and EdU suggested that over-expression of SEH1L significantly promoted the proliferation of HCC cells (Fig. 6A-C, F-G). Transwell experiment showed that SEH1L promoted the migration of Huh7 and PLC/PRF/5 cells (Fig. 6D-E). KEGG analysis has shown that SEH1L may participate in the regulation of ferroptosis, thus, we further detected the influence of SEH1L in GSH, malondialdehyde (MDA), mitochondrial membrane potential and ROS. The results suggested that over-expression of SEH1L increased the level of GSH, but decreased the level of MDA (Fig. 6H-I). Meanwhile, the mitochondrial membrane potential was up-regulated (Fig. 6J-K), but ROS was down-regulated (Fig. 6L-N).

**Fig. 7:**
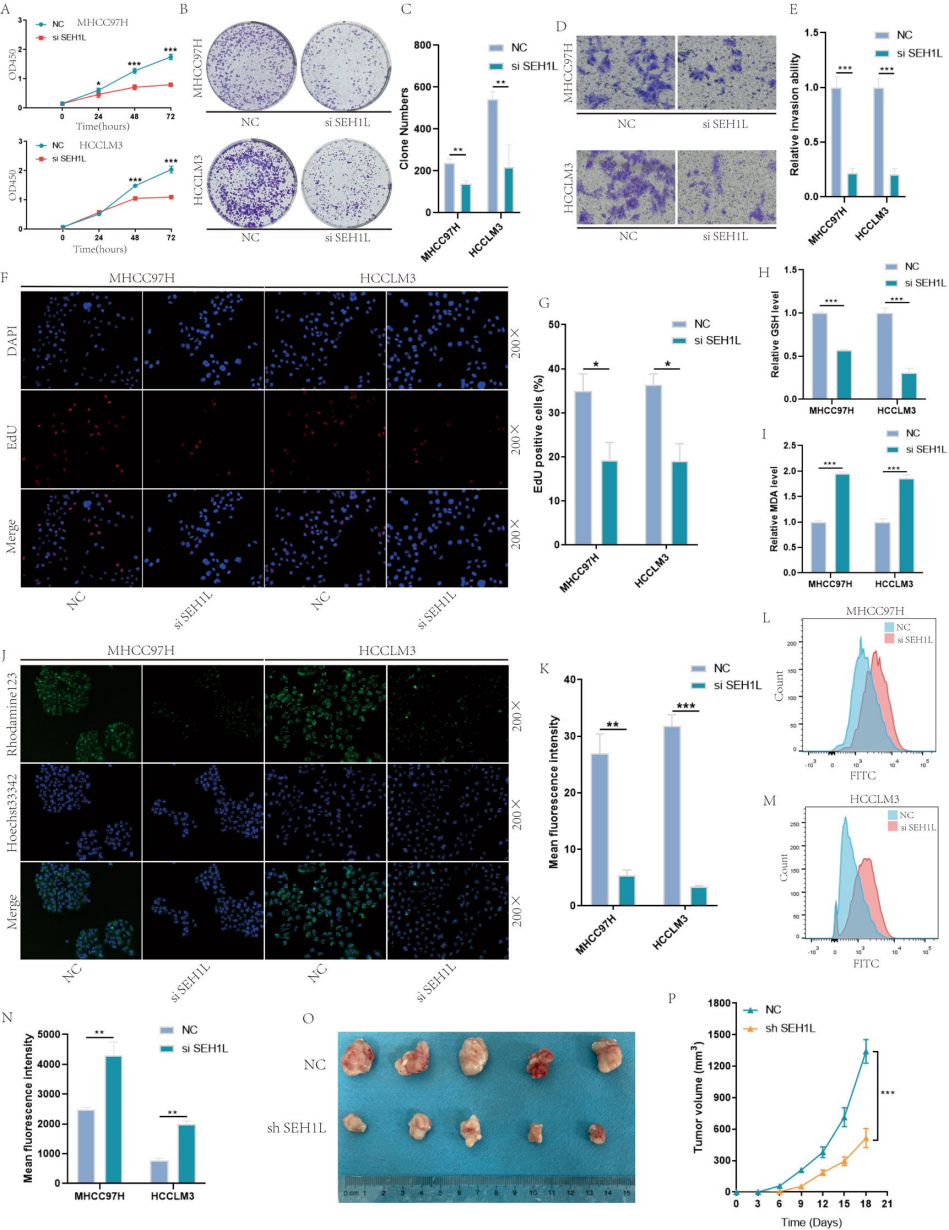
Knock down of SEH1L suppressed HCC progression. (**A**-**C**, **F**-**G**) Knock down of SEH1L suppressed the proliferation of HCC. (D-E) Knock down of SEH1L suppressed HCC migration. (**H**-**I**) Knock down of SEH1L decreased HCC GSH, but increased MDA. (**J**-**K**) Knock down of SEH1L decreased HCC mitochondrial membrane potential. (**L**-**N**) Knock down of SEH1L increased HCC ROS. (**O**-**P**) Knock down of SEH1L suppressed HCC progression in vivo. (* represents *P* < 0.05, ** represents *P* < 0.01, *** represents *P* < 0.001)

Similarly, knock down of SEH1L significantly suppressed the proliferation and migration of HCC cells (Fig. 7A-G). The results suggested that SEH1L siliencing significantly decreased the level of GSH, increased the level of MDA (Fig. 7H-I). Meanwhile, the mitochondrial membrane potential was down-regulated (Fig. 7J-K), but ROS level was significantly up-regulated (Fig. 7L-N). At last, we established the xenograft mouse models by subcutaneously injecting SEH1L knock down cells. As shown in Fig. 7O-P, the tumor of the SEH1L knock down group grew slower than that of the control group.

### SEH1L siliencing induced ferroptosis via ATF3/HMOX1/GPX4 axis

**Fig. 8:**
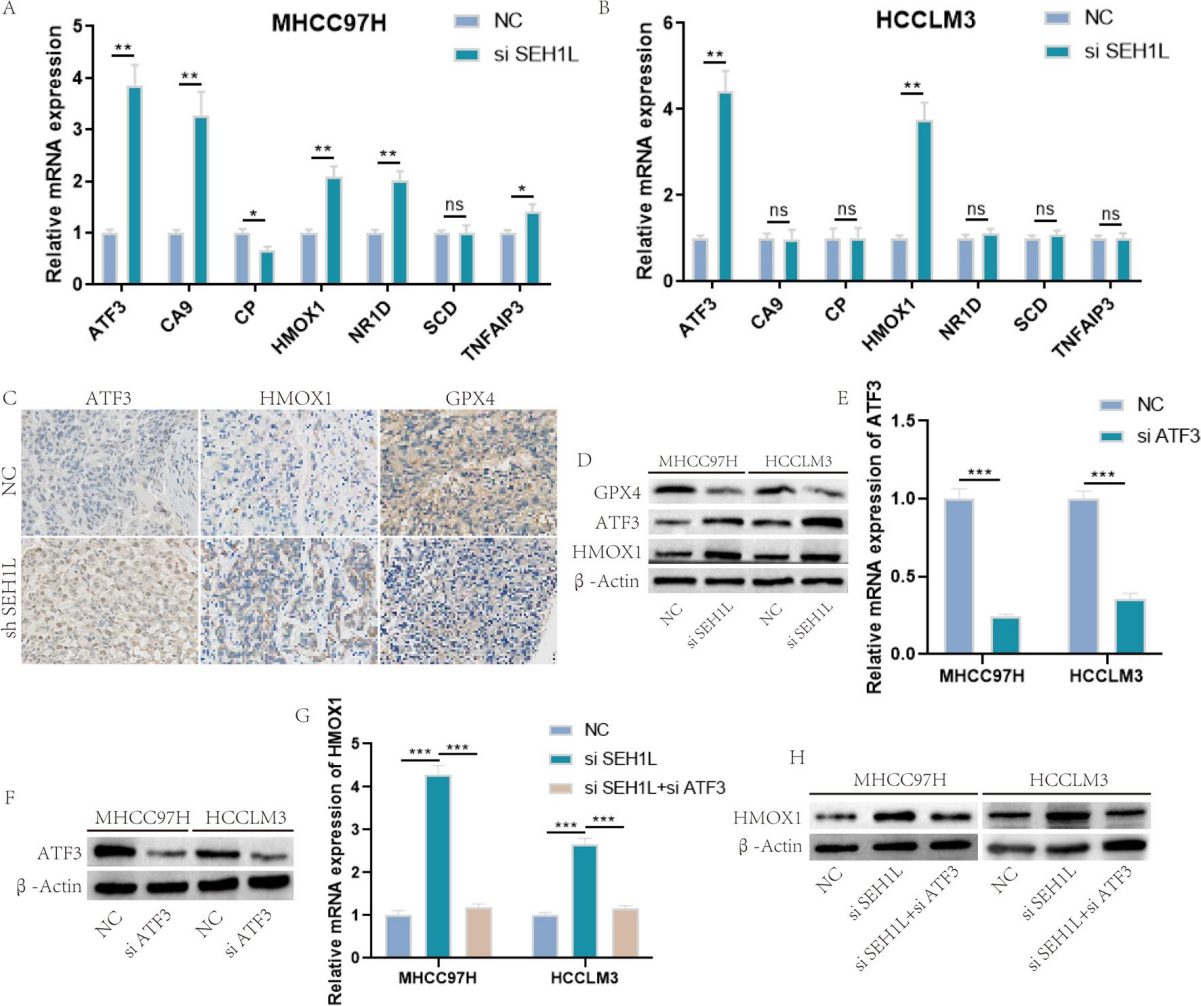
SEH1L siliencing activated ATF3/HMOX1/GPX4 pathway. (**A**-**B**) The mRNA level of ATF3 and HMOX1 were up-regulated after the knock down of SEH1L. (**C**-**D**) The protein level of ATF3, HMOX1 were up-regulated and GPX4 was down-regulated after the knock down of SEH1L. (E-F) The ATF3 knock down efficiency. (**G**-**H**) Knock down of ATF3 reversed the influence of SEH1L on HMOX1. (* represents *P* < 0.05, ** represents *P* < 0.01, *** represents *P* < 0.001)

Above results has suggested that knock down of SEH1L induced ferroptosis and suppressed HCC progression. Next, we further analyzed the next generation sequencing results and screened the genes in FerrDb V2, a database which collected all the ferroptosis related genes. At last, we identified 7 ferroptosis related genes, including ATF3, CA9, CP, HMOX1, NR1D, SCD and TNFAIP3 (sequencing fold change > 2). Then, the influence of SEH1L in these genes was validated using RT-qPCR. The results suggested that only ATF3 and HMOX1 was significantly up-regulated in both MHCC97H and HCCLM3 cells after the knock down of SEH1L (Fig. 8A-B). In recent years, ATF3/HMOX1/GPX4 pathway has been reported to induce ferroptosis, and ATF3 may function as a transcription factor of HMOX1 [26–28]. Thus, we further detected the expression of ATF3, HMOX1 and GPX4 using WB and IHC. The results suggested that ATF3, HMOX1 were up-regulated and GPX4 was down-regulated after the knock down of SEH1L (Fig. 8C-D).

**Fig. 9:**
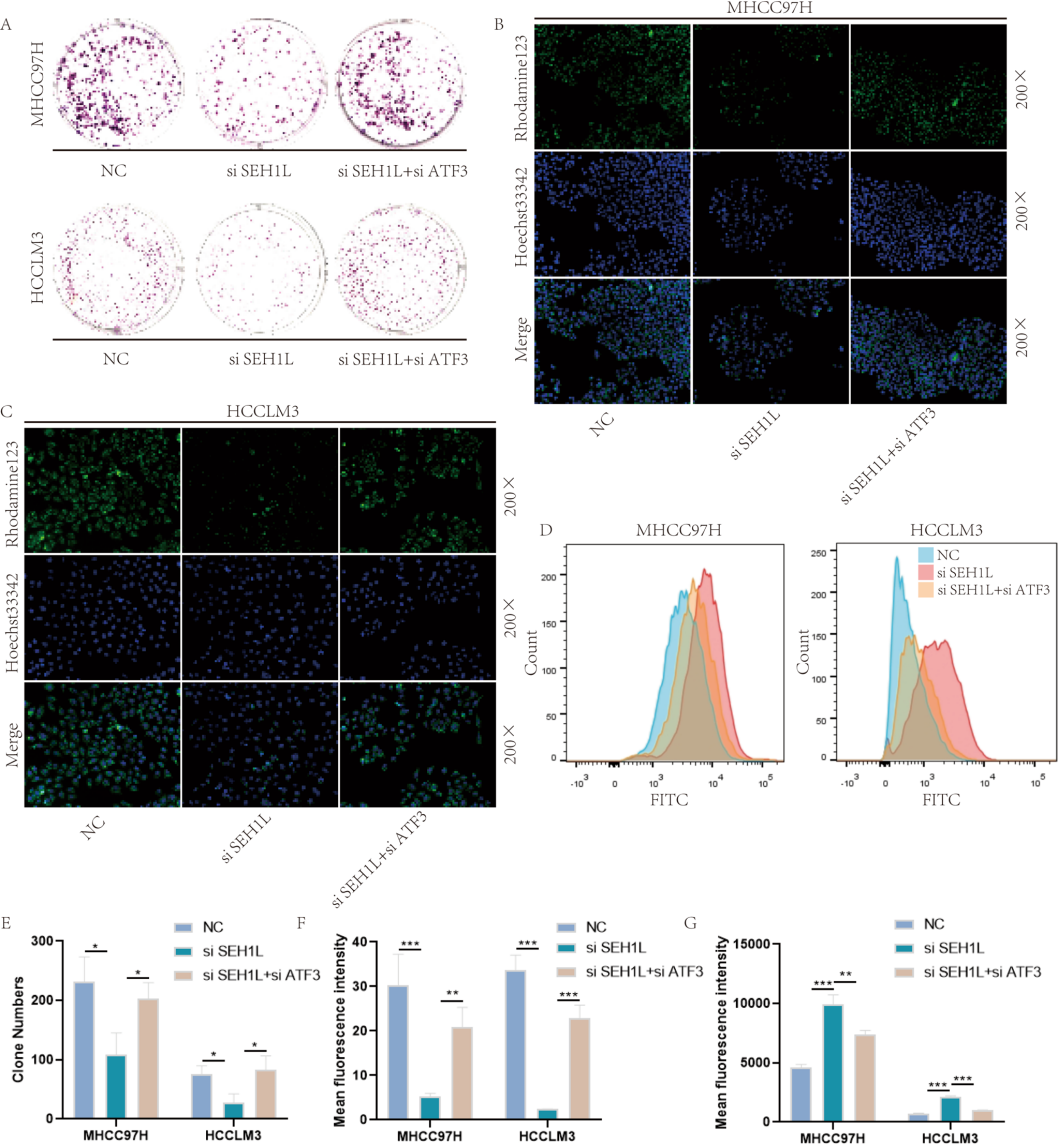
SEH1L siliencing induced HCC ferroptosis via ATF3/HMOX1/GPX4 axis. (**A**, **E**) Knock down of ATF3 rescued the influence of SEH1L on HCC proliferation. (**B, C, F**) ATF3 siliencing rescued the influence of SEH1L on HCC mitochondrial membrane potential. (**D, G**) ATF3 siliencing rescued the influence of SEH1L on HCC ROS. (* represents *P* < 0.05, ** represents *P* < 0.01, *** represents *P* < 0.001)

To further prove that SEH1L regulated ferroptosis through ATF3/HMOX1/GPX4 axis, we performed the rescue experiments. The knock down efficiency of ATF3 was shown in Fig. 8E-F. Besides, knock down of ATF3 could rescue the influence of SEH1L on HMOX1 (Fig. 8G-H). Furthermore, knock down of SEH1L could suppress proliferation (Fig. 9A, E), decrease mitochondrial membrane potential (Fig. 9B, C, F) and increase ROS (Fig. 9D, G), and all these effects could be reversed by knock down of ATF3.

## Discussion

In the past decades, many studies have focused on the function of SEH1L. At first, SEH1L was identified as a nucleoporin, and play significant roles in the formation of Nup107 complex [29]. Then, researchers found that SEH1L may also function as a component of GATOR2 complex and activate mTOR signalling [23]. Mei Wu et al. found that SEH1L could maintain the stability of genome by regulating the interaction between SETDB1 and KAP1, and knock out of SEH1L could induce the necroptosis of schwann cell [30]. What’s more, Stacie K. Loftus et al. found that HIF-1α could induce the expression of SEH1L and reduce time of disease free status in melanoma [31]. Based on previous work, we further analyzed the expression and potential function of SEH1L in pan-cancer. Our study showed that the expression of SEH1L was up-regulated in most cancers. High SEH1L expression was related to poor OS, DFI, PFI and DSS in LIHC. Meanwhile, the patients of UCEC with SEH1L mutation had better OS, DSS and PFS. What’s more, we further validated our bioinformatics result through in vitro and in vivo experiments. The RT-qPCR and WB experiments showed that SEH1L was significantly up-regulated in HCC tissues and cells. Besides, next generation sequencing suggested that SEH1L may participate in the regulation of ferroptosis. Besides, we found that knock down of SEH1L significantly induced ferroptosis and suppressed the progression of HCC via ATF3/HMOX1/GPX4 axis.

Above results are consistent with previous reports that ATF3/HMOX1/GPX4 axis could induce ferroptosis. Recently, ferroptosis has been identified as a new type of iron dependent cell death, which is different from apoptosis and autophagy. J Rao et al. found that ATF3 may function as a transcription factor and promote the expression of HMOX1, and then activate the ferroptosis of HCC [26, 27]. Shiyan Fu et al. found that PEGylated Cu2WS4 nanozyme induced ferroptosis by regulating the KEAP1/NRF2/HMOX1/GPX4 molecules [28]. Haiyingjie Lin et al. found that EF24, a synthetic analogue of curcumin, could promote the expression of HMOX1 and then suppress GPX4 to induce ferroptosil [32]. Our studies firstly showed that knock down of SEH1L may promote the expression of ATF3, and then result in the up-regulation of HMOX1 and down-regulation GPX4. Thus, SEH1L may function as a potential therapy target by regulating the ferroptosis of HCC.

However, there are still some limitations in our study. Although we found that knock down of SEH1L may down-regulate the mRNA level of ATF3, the mechanisms are still unclear and further investigation is necessary. Besides, expanding the clinical sample size may make the results more persuasive.

## Conclusion

SEH1L is up-regulated and is associated with poor prognosis in pan-cancer. SEH1L siliencing could induce ferroptosis and suppresses HCC progression via ATF3/HMOX1/GPX4 axis.

## Electronic supplementary material

Below is the link to the electronic supplementary material.

Supplementary Material 1

Supplementary Material 2

Supplementary Material 3

## Data Availability

The data are available from the corresponding author for reasonable requests.

## References

[1] Siegel RL, Miller KD (2023) Cancer statistics, 2023. CA Cancer J Clin 73:17–48 36633525 10.3322/caac.21763

[2] Li X, Ramadori P (2021) The immunological and metabolic landscape in primary and metastatic liver cancer. Nat Rev Cancer 21:541–557 34326518 10.1038/s41568-021-00383-9

[3] Qiu Z, Li H, Zhang Z, Zhu Z, He S, Wang X, Wang P, Qin J, Zhuang L, Wang W et al (2019) A Pharmacogenomic Landscape in Human Liver cancers. Cancer Cell 36:179–193e111 31378681 10.1016/j.ccell.2019.07.001 PMC7505724

[4] Donne R, Lujambio A (2023) The liver cancer immune microenvironment: therapeutic implications for hepatocellular carcinoma. Hepatology 77:1773–1796 35989535 10.1002/hep.32740 PMC9941399

[5] Komuta M, Ueno A, Sakamoto M (2023) The spectrum of primary liver cancers: heterogeneity and continuity. A foundation for diagnosis and treatment of cancer. Hepatology 77:10–12 35263454 10.1002/hep.32452

[6] Xu F, Jin T, Zhu Y, Dai C (2018) Immune checkpoint therapy in liver cancer. J Exp Clin Cancer Res 37:110 29843754 10.1186/s13046-018-0777-4 PMC5975687

[7] Carroll HK, Duffy AG, O’Farrelly C (2022) Liver Immunology, Immunotherapy, and Liver cancers: Time for a Rethink? Semin Liver Dis 42:212–224 35263795 10.1055/s-0042-1744143

[8] Palmer WC, Patel T (2012) Are common factors involved in the pathogenesis of primary liver cancers? A meta-analysis of risk factors for intrahepatic cholangiocarcinoma. J Hepatol 57:69–76 22420979 10.1016/j.jhep.2012.02.022 PMC3804834

[9] Lapis K, Johannessen JV (1979) Pathology of primary liver cancer. J Toxicol Environ Health 5:315–355 224201 10.1080/15287397909529752

[10] Marquardt JU, Andersen JB, Thorgeirsson SS (2015) Functional and genetic deconstruction of the cellular origin in liver cancer. Nat Rev Cancer 15:653–667 26493646 10.1038/nrc4017

[11] Foerster F, Gairing SJ, Müller L, Galle PR (2022) NAFLD-driven HCC: safety and efficacy of current and emerging treatment options. J Hepatol 76:446–457 34555422 10.1016/j.jhep.2021.09.007

[12] Yang C, Zhang H, Zhang L, Zhu AX, Bernards R (2023) Evolving therapeutic landscape of advanced hepatocellular carcinoma. Nat Rev Gastroenterol Hepatol 20:203–222 36369487 10.1038/s41575-022-00704-9

[13] Chen W, Chiang CL, Dawson LA (2021) Efficacy and safety of radiotherapy for primary liver cancer. Chin Clin Oncol 10:9 32576017 10.21037/cco-20-89

[14] Greten TF, Wang XW, Korangy F (2015) Current concepts of immune based treatments for patients with HCC: from basic science to novel treatment approaches. Gut 64:842–848 25666193 10.1136/gutjnl-2014-307990 PMC6311419

[15] Xu MJ, Feng M (2019) Radiation Therapy in HCC: what data exist and what data do we need to incorporate into guidelines? Semin Liver Dis 39:43–52 30536291 10.1055/s-0038-1676098

[16] Jiang X, Stockwell BR (2021) Ferroptosis: mechanisms, biology and role in disease. Nat Rev Mol Cell Biol 22:266–282 33495651 10.1038/s41580-020-00324-8 PMC8142022

[17] Li J, Cao F, Yin HL, Huang ZJ, Lin ZT, Mao N, Sun B, Wang G (2020) Ferroptosis: past, present and future. Cell Death Dis 11:88 32015325 10.1038/s41419-020-2298-2 PMC6997353

[18] Gao W, Wang X, Zhou Y, Wang X, Yu Y (2022) Autophagy, ferroptosis, pyroptosis, and necroptosis in tumor immunotherapy. Signal Transduct Target Ther 7:196 35725836 10.1038/s41392-022-01046-3 PMC9208265

[19] Xie Y, Kang R, Klionsky DJ (2023) GPX4 in cell death, autophagy, and disease. Autophagy 19:2621–2638 37272058 10.1080/15548627.2023.2218764 PMC10472888

[20] Miao Y, Chen Y, Xue F, Liu K, Zhu B, Gao J, Yin J, Zhang C, Li G (2022) Contribution of ferroptosis and GPX4’s dual functions to osteoarthritis progression. EBioMedicine 76:103847 35101656 10.1016/j.ebiom.2022.103847 PMC8822178

[21] Zheng Y, Wang Y, Lu Z, Wan J, Jiang L, Song D, Wei C, Gao C, Shi G, Zhou J et al (2023) PGAM1 inhibition promotes HCC Ferroptosis and synergizes with Anti-PD-1 Immunotherapy. Adv Sci (Weinh) 10:e2301928 37705495 10.1002/advs.202301928 PMC10582428

[22] He F, Zhang P, Liu J, Wang R, Kaufman RJ, Yaden BC, Karin M (2023) ATF4 suppresses hepatocarcinogenesis by inducing SLC7A11 (xCT) to block stress-related ferroptosis. J Hepatol 79:362–377 36996941 10.1016/j.jhep.2023.03.016 PMC11332364

[23] Bar-Peled L, Chantranupong L, Cherniack AD, Chen WW, Ottina KA, Grabiner BC, Spear ED, Carter SL, Meyerson M, Sabatini DM (2013) A tumor suppressor complex with GAP activity for the rag GTPases that signal amino acid sufficiency to mTORC1. Science 340:1100–1106 23723238 10.1126/science.1232044 PMC3728654

[24] Platani M, Santarella-Mellwig R, Posch M, Walczak R, Swedlow JR, Mattaj IW (2009) The Nup107-160 nucleoporin complex promotes mitotic events via control of the localization state of the chromosome passenger complex. Mol Biol Cell 20:5260–5275 19864462 10.1091/mbc.E09-05-0377 PMC2793300

[25] Yu J, Liu TT, Liang LL, Liu J, Cai HQ, Zeng J, Wang TT, Li J, Xiu L, Li N, Wu LY (2021) Identification and validation of a novel glycolysis-related gene signature for predicting the prognosis in ovarian cancer. Cancer Cell Int 21:353 34229669 10.1186/s12935-021-02045-0 PMC8258938

[26] Rao J, Qian X, Li G, Pan X, Zhang C, Zhang F, Zhai Y, Wang X, Lu L (2015) ATF3-mediated NRF2/HO-1 signaling regulates TLR4 innate immune responses in mouse liver ischemia/reperfusion injury. Am J Transpl 15:76–87 10.1111/ajt.12954 25359217

[27] Huang F, Shi X, Hu M, Yan H, Li X, Ding Y, Zheng X, Cai X, Dai S, Xia Q, Cai Y (2024) Blocking of FGFR4 signaling by F30 inhibits hepatocellular carcinoma cell proliferation through HMOX1-dependent ferroptosis pathway. Eur J Pharmacol 970:176493 38484925 10.1016/j.ejphar.2024.176493

[28] Fu S, Li Y, Shen L, Chen Y, Lu J, Ran Y, Zhao Y, Tang H, Tan L, Lin Q, Hao Y (2024) Cu(2) WS(4) -PEG nanozyme as multifunctional sensitizers for enhancing Immuno-Radiotherapy by Inducing Ferroptosis. Small :e2309537 10.1002/smll.202309537 38323716

[29] Belgareh N, Rabut G, Baï SW, van Overbeek M, Beaudouin J, Daigle N, Zatsepina OV, Pasteau F, Labas V, Fromont-Racine M et al (2001) An evolutionarily conserved NPC subcomplex, which redistributes in part to kinetochores in mammalian cells. J Cell Biol 154:1147–1160 11564755 10.1083/jcb.200101081 PMC2150808

[30] Wu M, Li M, Liu W, Yan M, Li L, Ding W, Nian X, Dai W, Sun D, Zhu Y et al (2023) Nucleoporin Seh1 maintains Schwann cell homeostasis by regulating genome stability and necroptosis. Cell Rep 42:112802 37453065 10.1016/j.celrep.2023.112802

[31] Loftus SK, Baxter LL (2017) Hypoxia-induced HIF1α targets in melanocytes reveal a molecular profile associated with poor melanoma prognosis. Pigment Cell Melanoma Res 30:339–352 28168807 10.1111/pcmr.12579 PMC5411287

[32] Lin H, Chen X, Zhang C, Yang T, Deng Z, Song Y, Huang L, Li F, Li Q, Lin S, Jin D (2021) EF24 induces ferroptosis in osteosarcoma cells through HMOX1. Biomed Pharmacother 136:111202 33453607 10.1016/j.biopha.2020.111202

