# Supplementary figures and images for: SEH1L siliencing induces ferroptosis and suppresses hepatocellular carcinoma progression via ATF3/HMOX1/GPX4 axis
Source: Apoptosis. 2024 Aug 2;29(9-10):1723–37. doi: 10.1007/s10495-024-02009-5 (PMC11416379; doi:10.1007/s10495-024-02009-5)

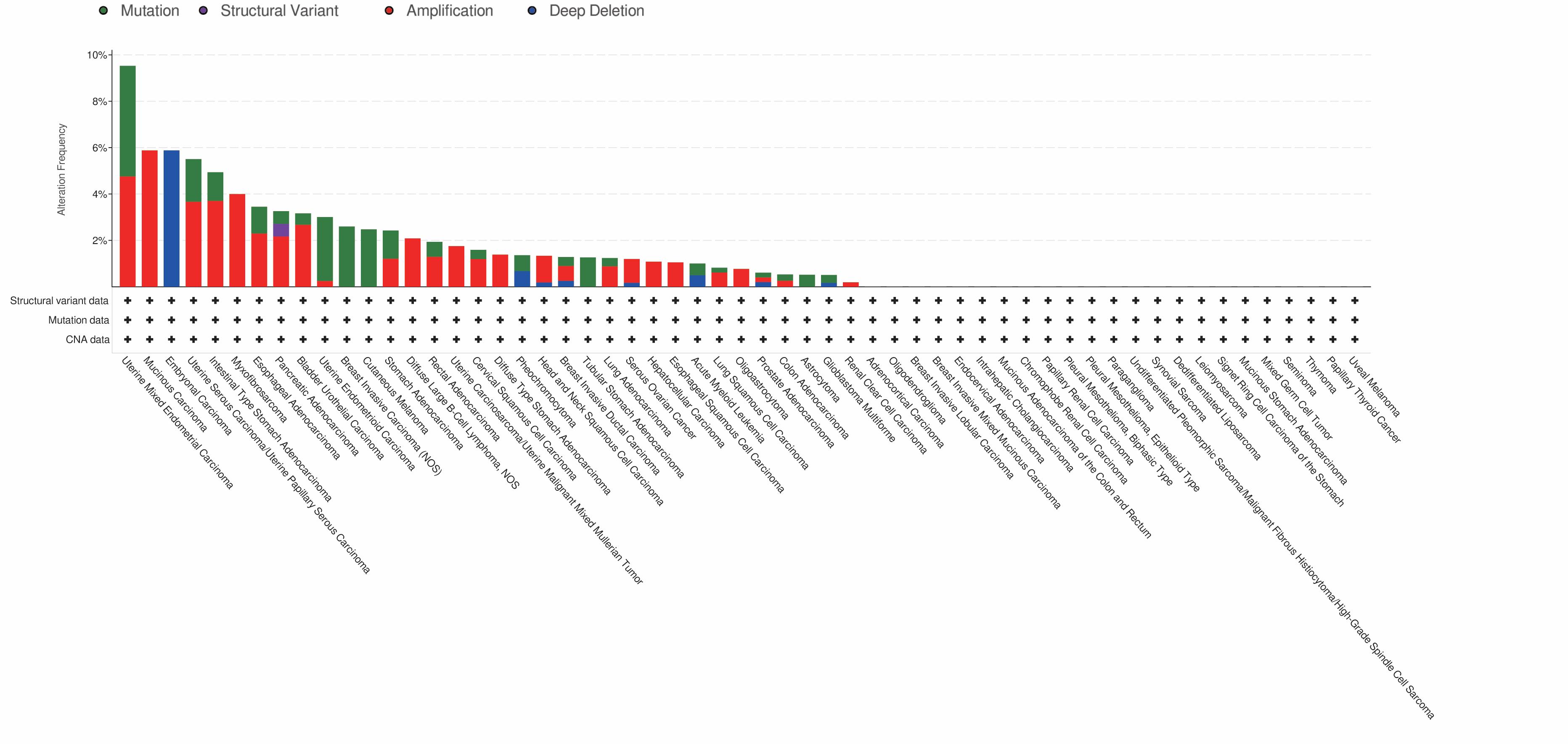

Supplement: Supplementary file 1 — Supplementary Material 1 [file 10495_2024_2009_MOESM1_ESM.jpg]

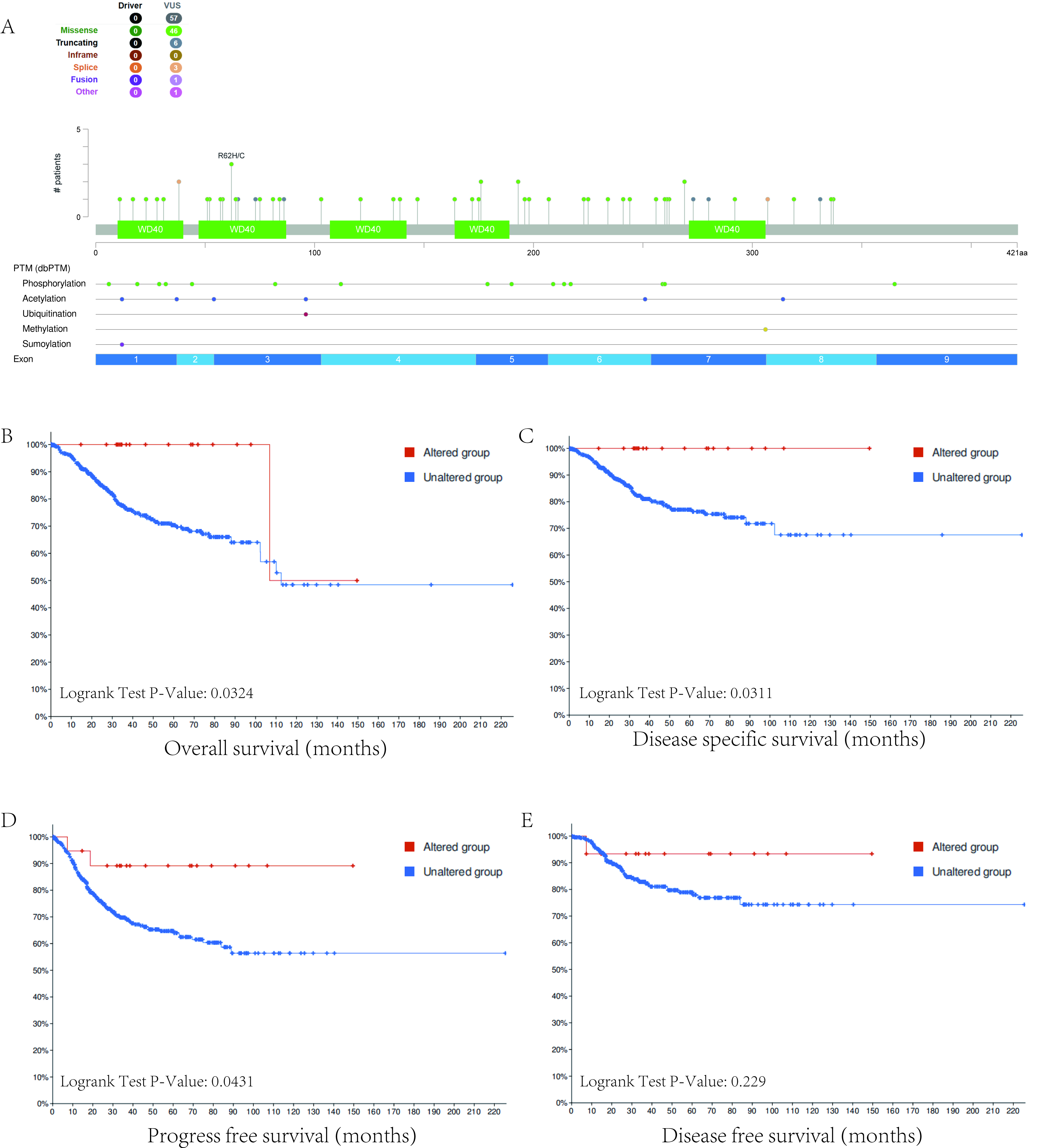

Supplement: Supplementary file 2 — Supplementary Material 2 [file 10495_2024_2009_MOESM2_ESM.jpg]
