# Supplementary material for: SEH1L siliencing induces ferroptosis and suppresses hepatocellular carcinoma progression via ATF3/HMOX1/GPX4 axis
Source: Apoptosis. 2024 Aug 2;29(9-10):1723–37. doi: 10.1007/s10495-024-02009-5 (PMC11416379; doi:10.1007/s10495-024-02009-5)
Supplement: Supplementary file 3 — Supplementary Material 3 [file 10495_2024_2009_MOESM3_ESM.docx]

**Figure S1. The alteration frequency of SEH1L in pan-cancer.**

**Figure S2. The mutation characteristic of SEH1L.** (A) The mutation subtypes and distributions of SEH1L. (B-E) OS, PFS, DFS and DSS analysis of SEH1L mutation status in UCEC cohort.
